# Epidural Analgesia for Pain Management in Acute Pancreatitis during Pregnancy and Its Effect on Maternal and Fetal Outcome

**DOI:** 10.1155/2022/3238613

**Published:** 2022-09-14

**Authors:** Sandeepika Dogra, Pallavi Sharma, Sunil Pandya, Manokanth Madapu, Soumya Jagannath Mahapatra, Ankita Sethi, Nilanchali Singh

**Affiliations:** ^1^Department of Anesthesiology and Critical Care, Fernandez Hospital, Hyderabad, India; ^2^Department of Obstetrics and Gynecology Government Medical College Kathua, Kathua, Jammu and Kashmir, India; ^3^Department of Anesthesiology and Critical Care, Fernandez Hospital, Hyderabad, India; ^4^Department of Gastro-enterology All India Institute of Medical Sciences Delhi, India; ^5^DM Reproductive Medicine Resident Department of Obstetrics and Gynecology All India Institute of Medical Sciences Delhi, Delhi, India; ^6^Gynecologic Oncology (Tom Baker Cancer Center-Canada) Department of Obstetrics and Gynecology All India Institute, of Medical Sciences Delhi, Delhi, India

## Abstract

**Background:**

Acute pancreatitis (AP) during pregnancy is a rare presentation with an estimated incidence of 1 case per 1000 to 10,000 pregnancies. Severe epigastric and abdominal pain is the earliest and the most common symptom of AP, and adequate pain relief is an integral part of patient management. The aim of our study was to investigate the different pain relief modalities that are used in pregnant women with AP and the efficacy of each method used, in terms of better pain relief and maternal-fetal outcomes.

**Methods:**

This was a retrospective observational study over a period of 6 years conducted at a tertiary care hospital. Pregnant women with clinical and biochemical diagnoses of acute pancreatitis were included in the study. Patient's history and clinical and biochemical data were collected from the medical records of the hospital.

**Results:**

A total of 12 patients were included in the study, 5 out of 12 patients had gall stones associated with AP, 2 patients had hypertriglyceridemia, and 1 each had preeclampsia and eclampsia. Epidural analgesia at the level of L1-L2 spinal level showed a reduction of VAS scores from 8 or 9 to 1 or 2, indicating excellent pain as compared to *t* intravenous (i/v) infusion of fentanyl or i/v boluses of injection tramadol, in whom VAS was never reduced below 3. With satisfactory results, labour analgesia and anesthesia for caesarean section was provided via the same catheter in 2 and 3 patients, respectively. Maternal and fetal outcomes were comparable in all the patients.

**Conclusion:**

AP in pregnancy, when diagnosed early and managed accordingly, leads to better maternal and fetal outcomes. Epidural analgesia was better than intravenous analgesia in terms of pain management and better recovery of acute pancreatitis patients. In these patients, labour analgesia and anesthesia for caesarean section can be provided through the same catheter, making it a potential novel modality in the treatment of acute pancreatitis in pregnancy.

## 1. Introduction

Acute pancreatitis (AP) during pregnancy is a rare presentation with an estimated incidence of 1 case per 1000 to 10,000 pregnancies [1–3]. The etiological causes of AP during pregnancy are similar to those in the general population. The most common risk factors are gallstone disease and hypertriglyceridemia. Gallstones are the most common cause of AP during pregnancy and are responsible for more than 70% of the cases. The spectrum of AP in pregnancy ranges from mild pancreatitis to severe pancreatitis associated with necrosis, abscess, pseudocysts, and multiple organ dysfunction syndromes (MODS). As with any other disease, AP has greater concerns during pregnancy.

The diagnosis of AP is more frequent in multiparous women (75%) [3, 4]. AP is rare during the first and second trimesters of pregnancy (12%), and it usually occurs in the third trimester (50%) or in the early postpartum period (35%) [3, 4, 5]. AP is more common with advancing gestational age, which parallels the incidence of gallstones in pregnancy. Over the past decade, due to significant improvements in diagnosis and maternal and neonatal intensive care, there has been a decrease in maternal and fetal mortality rates (from 37% to 60%, respectively) to 0–0.03% maternal mortality and 0.3% fetal mortality [1, 5, 9, 10]. Therefore, when properly managed, AP in pregnancy does not carry a dismal prognosis as in the past.

Severe epigastric and abdominal pain are the earliest and most common symptoms of AP, and adequate pain relief is an integral part of patient management [11]. Intravenous opioid analgesic is a commonly used modality for pain management in AP. Epidural analgesia causes excellent pain relief and is one of the most effective methods of perioperative pain management. However, the role of epidural analgesia in the management of AP is not very well defined, but many centers in the world have been using it for acute pain management in AP [12, 13]. One important reason why epidural analgesia is a better modality for AP pain management is that it causes improvement in pancreatic microcirculation due to sympathetic blockage and resulting vasodilatation, therefore preventing pancreatic necrosis. Pancreatic necrosis is an important cause of complications and poor prognosis in patients with acute pancreatitis; since epidural analgesia prevents pancreatic necrosis, it also prevents other adverse outcomes associated with it and ultimately decreases the morbidity associated with AP. Randomized placebo-controlled studies regarding the role of epidural analgesia in AP during pregnancy are lacking. A few studies are available that clearly show the beneficial effects of epidural analgesia in AP in nonpregnant populations [12, 13].

This is a retrospective study, which includes 12 cases of acute pancreatitis in pregnancy, who were admitted to our hospital from January 2013 to December 2018. The aim of our study was to investigate the different pain relief modalities used in AP in pregnancy and the efficacy of each method used, in terms of better pain relief and maternal-fetal outcomes.

## 2. Methodology

This is a retrospective observational study over a period of 6 years conducted in Fernandez Hospital, which is a recognized tertiary center for high-risk pregnancies and perinatology. The study was conducted from January 2013 to December 2018. Pregnant women with clinical signs and symptoms suggestive of acute pancreatitis were included in the study. Patient consent was obtained to use the patient data for research purposes. The diagnosis was confirmed using the revised Atlanta classification as defined by the presence of two of the following: (i) typical upper abdominal pain, (ii) increase in serum amylase and or serum lipase levels higher than three times the upper limit of normal, and (iii) imaging suggestive of AP. Abdominal ultrasound was done to see the pancreatic appearance, pancreatic duct, peripancreatic collection, and CBD and to detect gall stones in cases of AP. Fetal well-being was ensured with complete clinical and radiological evaluation. The severity and prognosis of AP were assessed by Ranson's criteria [14].

Conservative medical management was the mainstay of the treatment. In 7 out of 12 patients, intravenous (IV) analgesics (fentanyl and tramadol) were used for pain management. I.V. fentanyl was given as an infusion at a rate of 1 mcg/kg/hr, and tramadol was given as 100 mg i.v. boluses for 8 hourly intervals. In 5 patients, high lumbar epidural analgesia at the level of L_1_-L_2_ interspace was given, with boluses of 10–15 ml of 0.1% ropivacaine at 2 to 3 hours of intervals. The same dose is used via the same catheter for labour analgesia. Four out of 5 patients underwent preterm caesarean section in the epidural analgesia group, 10–12 ml of 0.2% ropivacaine via the same epidural catheter. The women were followed up until discharge. The APGAR scores and birthweight of neonates were noted at the time of delivery. Maternal and fetal conditions at discharge were noted.

### 2.1. Statistical Analysis

The data were collected in a Microsoft Excel sheet. The data were reported as numbers and percentages to characterize the patients' demographic and obstetrical parameters, results, and outcomes.

## 3. Results

Twelve patients with clinical and biochemical diagnoses of acute pancreatitis were included in the study. The mean age of the patients was 25.5 years, and 6 patients (50%) were in the age group of 25 to 30 years. None of the patients were below 20 years old, and only one patient was more than 35 years old, i.e., elderly gravida. Three-fourth of the patients was in their third trimester of pregnancy. Six patients were second-gravida, 3 were primigravida, and 1 patient was multigravida (parity >3). One patient had a bad obstetric history with 4 previous abortions. Demographic and obstetric parameters are described in Table 1. Five patients (41.7%) had gall stones associated with AP, 2 had hypertriglyceridemia, and 1 each was associated with preeclampsia and eclampsia, respectively, as shown in Table 2.

All the patients underwent transabdominal ultrasonography. Liver function tests, serum amylase, and serum lipase were done along with other baseline investigations, and the results are depicted in Table 3. Out of 12 patients, 2 (16.7%) had serum amylase level of more than 900 U/l, and serum lipase level of >900 U/l was found in 2 (16.7%) patients. Total bilirubin of >2 mg/dl was found in 2 (16.7%) patients. ALT and AST levels were not increased beyond significant levels (>70U/L) in most of the patients. Two patients were given an I/V infusion of fentanyl at a rate of 1 mcg/kg/hr. VAS scores in these two patients decreased to 3. Five patients (47%) were given 100 mg I/V boluses at 6 hourly intervals. In these patients, VAS scores were not reduced beyond 4. In 5 patients, high lumbar analgesia was provided after inserting the epidural catheter in lumbar interspace L1-L2. These patients experienced a reduction of VAS scores from 8 or 9 to 1 or 2, indicating excellent pain relief after boluses of 10–15 ml of 0.1% ropivacaine at 2-hour intervals, as shown in Table 4. All the patients delivered within 48–72 hours of hospital admission and therefore required epidural analgesia for a maximum of 72 hours.

Eight out of 12 (66.7%) patients had preterm delivery (<37 weeks), and all patients underwent LSCS, with no other maternal complications. In the fentanyl group, out of 2, 1 baby required NICU care because of neonatal jaundice. In the tramadol group, out of 5 patients, 3 had preterm deliveries out of which 1 baby required NICU care because of respiratory distress. In the epidural analgesia group, out of 5 patients, 4 had preterm deliveries, and 1 baby required NICU care in view of neonatal jaundice, as shown in Table 5. The average birthweight of the neonates was 2782 g. The majority of the babies had an APGAR >8, as shown in Table 6.

## 4. Discussion

Acute pancreatitis (AP) in pregnancy is an uncommon presentation. Almost 80% of the cases are mild, and only 20% of the cases are moderate to severe. It is most often associated with gallstone disease or hypertriglyceridemia [1, 2]. Gallstones are the most common cause of acute pancreatitis during pregnancy, accounting for more than 70% of the cases. Cholesterol secretion and hepatic bile secretion increased in the second and third trimesters as compared to bile and phospholipids, which leads to supersaturated bile and thus gallstone formation. In addition, fasting and postprandial gallbladder volumes are greater with a reduced rate of emptying. This large residual volume of supersaturated bile in the gallbladder leads to cholesterol crystals and eventually gall stones. Five of our patients had gall bladder stones. A second common scenario noted in pregnancy is hypertriglyceridemia-induced pancreatitis, and 2 of our patients had pancreatitis due to the same. Hypertriglyceridemia can be attributed to increased estrogen in pregnancy and the familial tendency of some women towards high triglyceride levels [15]. One patient each had preeclampsia and eclampsia, respectively.

Acute pancreatitis in pregnancy is more difficult to diagnose in the first trimester as compared to the second and third trimesters because the signs and symptoms mimic hyperemesis gravidarum. The majority of our patients presented with epigastric pain, anorexia, nausea, and vomiting. Therefore, in pregnant women presenting with severe nausea and vomiting, we should always evaluate the serum amylase and lipase levels. None of our patients had associated pulmonary findings. Abdominal tenderness and guarding were found in 50% of the patients. Two patients presented with jaundice with increased total bilirubin and AST levels. Abdominal ultrasound was done in all the cases as it is more sensitive for the diagnosis of acute pancreatitis and is safer than CT in pregnancy.

The management of AP in pregnancy does not vary from that in a nonpregnant state. It includes fluid restoration, oxygen supplementation, analgesics, and cessation of oral feeding to suppress the exocrine function of the pancreas [16]. Conservative management is the preferred therapeutic method, in particular, for mild acute pancreatitis [17]. All the patients in our study were managed conservatively, which included prophylactic antibiotics along with I/V fluids, oxygen, and analgesia. The role of antibiotics in the mild form of AP remains controversial. A systematic review and meta-analysis showed antibiotic prophylaxis does not reduce the mortality or protect against infected necrosis and frequency of surgical intervention [18].

One of the most important symptoms of AP is epigastric and abdominal pain. Therefore, pain management in AP patients is of prime importance. The most common mode of providing analgesia in AP patients is via intravenous opioids. Epidural analgesia is primarily used by anesthesiologists to treat pain in the postoperative period and for obstetric analgesia [19]. There has been recent interest in the use of epidural analgesia as a treatment for AP, and growing evidence from experimental studies now supports the beneficial effects of epidural analgesia that include increased mucosal capillary perfusion, increased gut barrier function, increased renal perfusion, and decreased severity in addition to profound analgesia. Bernhhardt et al. gave epidural analgesia to 121 patients, where excellent analgesia (VAS <2) was achieved in 72% of the patients. Sadowski et al. did a comparative study which showed TEA (thoracic epidural analgesia) and showed better pain management with TEA and increased pancreatic microcirculation. In our study, patients who received high lumbar epidural analgesia VAS scores decreased from 9 or 8 to ≤2, indicating profound pain relief. All patients were in their third trimester and delivered via emergency caesarean section. Labour analgesia was provided in 2 of these patients via the same epidural catheter, and the results were satisfactory. All patients who underwent emergency caesarean section in the epidural analgesia group received anesthesia through the same epidural catheter with a dose of 10–12 ml of 0.2% ropivacaine with good surgical conditions. Since all patients were delivered within 48–72 hours of admission, we did not need to give epidural analgesia beyond 72 hours. However, if the labour has to go beyond this period, further management of pain with the help of pain specialists is warranted. There was no maternal or fetal mortality in any of these 12 patients. However, 1 baby from each group had to be shifted to NICU, 2 because of neonatal jaundice, and 1 because of respiratory distress owing to prematurity and low birth weight. This shows that the maternal and fetal outcomes were comparable in all three groups. One of the authors has published an article on maternal and fetal outcomes in pancreatitis and reported no increased risk of maternal and fetal outcomes [21].

The limitations of this study are its retrospective nature and very small sample size. Yet, the strength is that it focuses upon a cohort of patients in which analgesia is the mainstay of management.

## 5. Conclusion

Epidural analgesia is better than conventional intravenous analgesia in pregnant women with acute pancreatitis, as it also aids in improving disease prognosis. In these patients, labour analgesia and anesthesia for caesarean section along with pain management of AP can be provided through the same catheter when required, which makes it a potential novel modality in the treatment of acute pancreatitis in pregnancy. Large-scale studies are necessary to validate these results.

## Figures and Tables

**Table 1:** Demographic and obstetric profile of the patients.

Parameter	Range	Number and percentage
Age	20–25 years	5 (41.7%)
	26–30 years	6 (50%)
	30–35 years	0 (0%)
	>35 years	1 (8.3%)

Period of gestation	First (till 12 weeks)	0 (0%)
	Second (13–28 weeks)	3 (25%)
	Third (29–40 weeks)	9 (75%)

Gravida	Primigravida	3 (25%)
	Parity: 1–2	6 (50%)
	Parity: 2–3	2 (16.7%)
	Parity: >3	1 (8.3%)

Abortions	*A*_0_	8 (66.7%)
	*A*_1_	3 (25%)
	*A*_2_	0 (0%)
	*A*_3_	1 (8.3%)

**Table 2:** Risk factors associated with acute pancreatitis in pregnancy.

Risk factors	Number and percentage
(1) Gall bladder stones	5 (41.7%)
(2) Hypertriglyceridemia	2 (16.7%)
(3) Preeclampsia	2 (16.7%)
(4) Eclampsia	1 (8.3%)
(5) Hypercalcemia	1 (8.3%)
(6) Idiopathic	2 (16.7%)

**Table 3:** Baseline investigations of the pregnant patients with acute pancreatitis.

S. no.	Biochemical parameters	Number of patients	Percentage
1	S-amylase (U/L) 100–300 300–600 600–90 900	3 4 3 2	25 33.4 25 16.7

2	S-lipase (U/L) 100–300 300–600 600–900 >900	4 3 3 2	33.4 25 25 16.7

3	Total bilirubin (mg/dl) 0.2–1.3 1.3–2.0 >2.0	9 1 2	75 8.3 16.7

4	Serum AST (U/L) 10–40 40–70 >70	9 2 1	75 16.7 8.3

5	Serum ALT ((U/L) 10–40 40–70 >70	10 2 0	83.3 16.7 0

**Table 4:** Change in VAS scores of patients with respect to the mode of analgesia.

I.V. fentanyl infusion	I.V. tramadol boluses	Epidural analgesia
P_1_: 8⟶3 P_2_: 9⟶3	P_1_: 9⟶4	P_1_: 9⟶1
	P_2_: 8⟶5	P_2_: 8⟶2
	P_3_: 8⟶5	P_3_: 9⟶2
	P_4_: 8⟶4	P_4_: 8⟶2
	P_5_: 7⟶3	P_5_: 8⟶1

P: patient; VAS: Visual Analog Score.

**Table 5:** Maternal and fetal complications in different analgesia groups.

I.V. fentanyl (*n* = 2)	I.V. tramadol (*n* = 5)	Epidural analgesia (*n* = 5)
Maternal complications: preterm C-sec; 1 fetal complications: prematurity (1)	Maternal complications: preterm vaginal delivery (1) and preterm C-sec (2). Fetal complications: prematurity (3); small for gestational age (1); respiratory distress (1); NIV for 2 days	Maternal complications: preterm VD (1); preterm CS (3). Fetal complications: prematurity (4); neonatal jaundice (1)

CS: caesarean section—full-term caesarean delivery; VD: assisted vaginal delivery; NIV: noninvasive ventilation; RD: respiratory distress; NNJ: neonatal jaundice; NNH: neonatal hypoglycemia.

**Table 6:** Neonatal outcomes of the study group.

S. no.	Period of gestation	Mode of delivery	Birthweight	APGAR score
1	36 + 2 weeks	LSCS	Girl, 3200 gm	8/8/9
2	36 + 2 weeks	LSCS	Boy, 3040 gm	8/8/9
3	38 + 2 weeks	LSCS	Girl, 1880 gm	8/9/9
4	40 weeks	LSCS	Boy, 4200 gm	8/8/9
5	40 weeks	LSCS	Boy, 2960 gm	7/8/9
6	38 + 6 weeks	LSCS	Boy, 3880gm	8/8/9
7	40 + 3 weeks	LSCS	Girl, 3000gm	8/9/9
8	35 + 2 weeks	LSCS	Boy, 2800 gm	8/8/9
9	41 weeks	LSCS	Girl, 3760 gm	8/9/9
10	26 + 2 weeks	LSCS	Girl, 750 gm	0/3/5
11	30 weeks	LSCS	Boy, 1809 gm	7/8/9
12	32 + 1 weeks	LSCS	Girl, 1909 gm	8/9/9

## Data Availability

The data used to support the findings of this study are available from the corresponding author upon request.
